# Perceptions and attitudes towards companion animal brain banking in pet owners: A UK pilot study

**DOI:** 10.1002/vro2.36

**Published:** 2022-05-28

**Authors:** Thomas J. A. Cardy, Daniel Jewth‐Ahuja, Abbe H. Crawford

**Affiliations:** ^1^ Cave Veterinary Specialists, George's Farm West Buckland Wellington UK; ^2^ Clinical Science and Services Royal Veterinary College AL9 7TA, North Mymms, Hatfield Herts UK

## Abstract

**Background:**

Detailed analysis of archived brain tissue is fundamental to advancing the understanding of neurological disease. The development of the UK Brain Bank Network (UBBN) has provided an invaluable resource to facilitate such research in the human medical field. Similar resources are needed in veterinary medicine. However, collection and archiving of companion animal brain tissue is a potentially sensitive area for pet owners and veterinary professionals.

**Methods:**

Using an online survey, we aimed to study pet owners’ perceptions of brain banking. The survey included information on respondents, their views on organ donation, the UBBN and the Royal Veterinary College's Companion Animal Brain Bank (RVC CABB).

**Results:**

In total 185 respondents were included. The use of brain tissue from pets for research was supported by 87% of respondents, and 66% of respondents felt that they were highly likely or likely to donate their pet's brain tissue to a CABB. Furthermore, 94% felt that more information on tissue banking in companion animals should be readily available.

**Conclusions:**

We found that the perceptions of companion animal brain banking were positive in our respondents. Open dialogue and clear information provision on the process and benefits of the CABB could enhance awareness and thus facilitate brain donation for translational research.

## INTRODUCTION

Neurological conditions negatively impact the quality of life of humans and companion animals, resulting in significant challenges for the affected individuals, their carers and owners. There are over 600 human neurological conditions recognised in the UK and, as the population increases in size along with increases in average age and life expectancy, there is a concomitant increase in neurological disease burden, with approximately 45 million cases of brain disease per year in the UK.^1^, ^2^ Consequently, there is a need for high‐quality, large‐scale neuroscience research to provide novel treatments and improved management strategies.^3^ Despite advances in imaging modalities, computational neuroscience and functional genomics, much is unknown about neurological diseases at the cellular and molecular levels. In the last 30 years significant advances in neurological disease research have come from the detailed examination of human and animal brain tissue.^4^, ^5^

Globally, there is an acknowledged paucity of brain tissue available to researchers. In human medicine this resource gap is being successfully bridged by the creation of the UK Brain Bank Network (UBBN), which ensures that human brain tissue is readily accessible to researchers from any discipline.^6^ Brain banks are facilities that store high‐quality postmortem brain and spinal cord tissue donated by patients with neurological disease, as well as healthy individuals.^7^, ^8^ In addition to stored tissue, biological fluids including whole blood, serum, plasma, urine and cerebrospinal fluid (CSF), are archived. There are currently 10 brain banks across the UK storing brain tissue from patients with neurological disease, with an additional two banks set up solely for the collection of healthy tissue to act as control samples for comparative studies. More than 12,000 donated brains have been catalogued across the UK. All banks utilise unified protocols for consent, donation, tissue handling and storage as well as for the application process for tissue sample acquisition.^6^, ^9^

Utilising the methodologies and expertise from the UBBN, the UK's first Companion Animal Brain Bank (CABB) was established at the Royal (Dick) School of Veterinary Studies.^10^ A standardised methodology for collection was adapted from the Medical Research Council's Brain Bank Network to ensure systematic sample collection, processing, storage and cataloguing of brain tissue. Furthermore, utilising methodology from human medicine ensures that brain areas of interest are directly comparable between species. Cats and dogs have a range of spontaneously occurring diseases similar to those of humans, such as age‐related cognitive decline, gliomas and epilepsy.^11^ The prevalence of cognitive dysfunction syndrome was reported to be 22.5% in dogs older than 9 years and 68% in 15−16 years old dogs.^12^, ^13^ The incidence of brain tumours in adult dogs has been reported to be 2.8%–4.5%, with gliomas representing 36%–70% of primary brain tumours in dogs.^14^, ^15^, ^16^, ^17^ The prevalence of idiopathic epilepsy in dogs has been estimated at 0.6%–0.75% in the general canine population,^18^, ^19^ while a study of recurrent seizure disorders in cats presented to primary care practices in the UK reported a 1‐year period prevalence of 0.16%.^20^ Thus, detailed analysis of feline and canine brain tissue can increase our understanding of such diseases and facilitate advances in both veterinary and potentially human medicine.^21^ Based on these protocols, a CABB was established at the Royal Veterinary College (RVC) in October 2018 to provide an equivalent archive. The RVC CABB is currently collecting and archiving brain tissue and biological fluids (whole blood, serum, plasma, urine and CSF) from dogs and cats euthanised as a result of neurological disease. The bank also stores tissues from appropriate controls euthanised due to non‐neurological conditions. At the time of writing, 42 brains have been donated by owners and archived in the RVC CABB (for further information, see Supporting Information S1).

Public perception of brain and tissue banking and of the willingness to consent to donate has been studied in human medicine and varies widely according to multiple factors including ethnicity, religion, education and socioeconomic status.^22^ Perceptions of companion animal brain banking and the barriers to pet brain and organ donation remain unknown. Therefore, the aim of this study was to assess pet owner perceptions of brain and tissue banking via an online questionnaire to increase awareness of the existence of the CABB and to optimise the consent and donation process for companion animals.

## MATERIALS AND METHODS

### Questionnaire design

An online questionnaire was hosted on SurveyMonkey (SurveyMonkey Europe, Shelbourne Road, Dublin, Ireland) from 26 April 2018 to 20 August 2018. Owners of dogs were recruited via social media including the Facebook pages ‘New Faces at the RVC’ and ‘Royal Veterinary College Canine Epilepsy Research’ and the RVC Twitter page. All three social media pages are publicly accessible nationally and internationally but primarily reach clients, staff, students and others affiliated with the institution. Study author contact details were included in the advertisement. Participant consent was obtained via a statement at the start of the questionnaire, and the study was approved by the RVC's Ethics and Welfare Committee (approval number SR2017‐1327). The survey consisted of a total of 42 questions divided into five sections. The design was influenced by previous questionnaires on human organ donation and perceptions of animal research.^5^, ^23^, ^24^ Key sections are summarised below, and the full questionnaire is available as Supporting Information S2.

#### Introduction

Background information was provided, including a summary of the current limitations surrounding the availability of high quality tissues for neurological research, the establishment of brain banking facilities and the goals of the RVC's CABB. An overview of the consent and donation process was also provided (Supporting Information S3 and S4).

#### Part 1: Owner information

Nine questions on general client information included age, gender, religion, occupation, country of residence, level of education/qualifications, pet ownership, number and date of prior visits to RVC's Queen Mother Hospital for Animals (QMHA).

#### Part 2: Companion animal details

Thirteen questions included the canine/feline's age, breed, sex, spay/neuter status, current disease history and current or previous neurological signs, including seizures and behaviour changes. Additionally, participants were asked about their knowledge of neurological diseases affecting their pet's breed.

#### Part 3: Views of participants on organ donation in humans

Five questions on participant perspectives on human organ donation, including which organs they support donation of, whether they themselves were on the NHS Donor Register and whether they support utilising human organs for research purposes.

#### Part 4: Introduces the UBBN and research into veterinary neurological conditions

Six questions utilising a five‐point Likert scale and ‘yes’/’no’ checkboxes. This section establishes prior knowledge of the UBBN, whether participants personally know anyone with a neurological disorder and the usage of animal tissues in veterinary and human disease research.

#### Part 5: Overview of the RVC CABB and of the donation process

Nine questions used a Likert scale to ascertain support for the CABB project and whether the participant would give consent to donate their pet's brain. The reasons for and against giving consent were investigated using free text and drop‐down lists. This section also investigated when the best time would be to provide consent to donate and the preferred manner of receiving information about the CABB.

### Data analysis

Survey responses were collated and summarised descriptively. Percentages were calculated from the total number of responses obtained for each question. The respondents were then separated into those affiliated with the veterinary profession (e.g., veterinary students, veterinary surgeons, veterinary nurses or animal/patient care assistants) and those not affiliated with the veterinary profession. Responses were quantified for each subgroup and compared statistically using Fisher's exact or chi‐squared tests (SPPS Software, Version 28). A *p*‐value <0.05 was considered statistically significant.

## RESULTS

### Included respondents

During the 4‐month period that the online survey was accessible, a total of 186 responses were received. One participant did not complete the consent statement and so their data were excluded from the analysis. Answers to all survey questions were provided by 144 participants (78%) and 41 participants (22%) omitted one or more responses. Where a response was not provided this is stated in the text.

### Respondent profile

Summary data of the responder demographic profile are shown in Table 1. One hundred and seventy‐two respondents were female (93%), 12 (7%) were male and one identified as other. Sixty‐two percent of respondents were aged between 18 and 34 years, with only 10% of respondents over the age of 55 years. The majority of respondents were from the UK (178, 96%). Most respondents identified as atheist (72, 39%) or Christian (66, 36%). Over 34 different occupations were represented, with students representing the single largest responder group (58, 31%). Eighty‐seven (47%) responders were involved in the veterinary professions as either veterinary students (48, 26%), veterinary surgeons (20, 11%), veterinary nurses (17, 9%) or patient care assistants (2, 1%). Ninety‐seven respondents (53%) had completed a degree or postgraduate course.

**TABLE 1:** Summary of the key demographics of the 185 questionnaire respondents

Demographic	Number of respondents
Sex	
Male	12
Female	172
Other	1
Age (years)	
18–34	114
35–54	53
55+	18
Country of residence	
UK	178
Other	7
Religion	
Atheist	72
Christian	66
Agnostic	27
Other	12
Profession	
Veterinary student	48
Veterinary surgeon	20
Veterinary nurse or patient care assistant	19
Researcher	6
Other	92
Pet(s) owned	
Dog	137
Cat	64
Exotic	31
Equine/livestock	7

All respondents had pets, with the majority (137, 75%) being dog owners, 64 (35%) owned cats, 31 (17%) owned exotic animals and a further seven (4%) owned horses or livestock. Of the 137 respondents who answered the questions, 44 (32%) had visited the QMHA with their pet in the previous 10 years. When considering their current pet's health, 58 (42%) respondents stated that their pet was suffering from an illness, including neurological (23, 17%) or musculoskeletal conditions (10, 7%). Fifty‐two (38%) respondents were aware of pets with neurological conditions belonging to people they knew personally, and 33 (24%) respondents were aware of neurological conditions that affect their pet's breed, with epilepsy (18, 13%) being the most well‐known.

### Perceptions of human organ donation

Of the 145 respondents who answered the questions, 130 (90%) were supportive of human organ donation, three (2%) respondents did not support human organ donation and 12 (8%) respondents were unsure if they supported the process or not. One hundred one (70%) respondents were on the NHS organ donor register, and 123 (85%) respondents were strongly in support of donating human organs for research. One hundred and nineteen respondents (82%) were in favour of the ‘Opt‐out’ scheme for human organ donation, where organ donation is routinely carried out unless explicitly specified by the patient's family.

### The UK Brain Bank Network and veterinary neurology

Of the 144 respondents who answered the questions, 124 (86%) were not aware of the existence of the UBBN prior to this survey, but 122 (85%) knew of a person who had been diagnosed with a brain disease. Of the 143 respondents who answered the questions, 94 (66%) were aware of brain tissue from pets being used for human and veterinary research. Seventy‐two (50%) respondents were strongly supportive of brain tissue from pets being used in research, with a further 53 (37%) being supportive. Four (3%) respondents either strongly disagreed or disagreed with brain tissue from pets being used in research.

### Royal Veterinary College's Companion Animal Brain Bank

Of the 135 respondents who completed the questions, the RVC CABB initiative was strongly supported by 74 (54%) respondents, with an additional 49 (36%) supporting the project. Three (2%) respondents either strongly opposed or opposed the project. These three respondents also strongly opposed the use of animal tissue for any sort of human or veterinary research. Thirty‐four (25%) and 48 (36%) respondents thought that the existence of the RVC CABB would either strongly positively or positively influence perceptions of the institution, respectively. Sixty‐seven (50%) and 55 (41%) respondents thought that the RVC CABB project would benefit human and veterinary research, respectively.

### Brain donation and factors driving decision making

Ninety (66%) respondents felt that they were either highly likely or likely to donate their pet's brain tissue to the brain bank. Eighteen (14%) respondents felt highly unlikely or unlikely to donate their pet's brain tissue. Of the 132 respondents who answered the questions, the main reasons for supporting the CABB included the thought of their pet being generally beneficial to neuroscience research (93, 71%) and specifically improving veterinary neuroscience research (72, 55%). Other comments emphasised the desire to contribute to further research at a more personal level, as respondents were aware of an individual (61, 46%) or pet (44, 33%) with a neurological condition. Conversely, of the 82 respondents who answered the question, reasons for being less likely to give consent included the inability to bury or cremate their pet intact (35, 43%), unwillingness for procedures to take place on their pet after its death (33, 40%) and their pet not being able to give consent to the procedure (23, 28%). Seven respondents (9%) felt inadequately informed to go ahead with the procedure despite reading the provided summary information on the CABB.

### Provision of consent and information on companion animal tissue donation

Of the 132 respondents who answered the question, 53 (40%) were in favour of providing consent for tissue donation at the time of diagnosis of a neurological condition where the pet's health is deteriorating, 30 (23%) felt that provision of consent should be at the time of euthanasia and 22 (17%) when the pet is still in good health. The remainder felt that the timing of consent would vary on the circumstances and diagnosis.

Of the 126 respondents who answered the question, 85 (67%) were happy to speak to their primary veterinary surgeon regarding donation to a brain bank, 80 (64%) felt comfortable using online resources, 70 (56%) were happy to receive direct emails and 57 (45%) felt that information was best provided via literature in the referral veterinary hospital. In addition, 118 (94%) respondents felt that more information on the UBBN and tissue banking in companion animals should be readily available to the general public.

### Comparison of veterinary‐affiliated and non‐veterinary‐affiliated respondents

**FIGURE 1:**
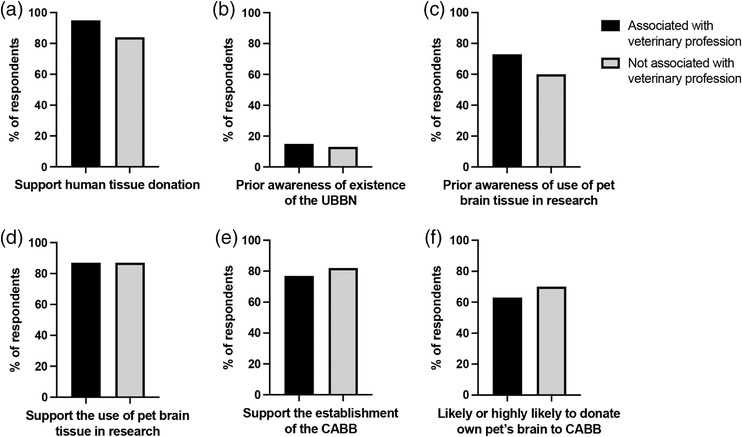
Summary of comparison of responses to key questions between respondents associated with the veterinary profession and those not associated with the veterinary profession. No significant differences were identified in the number of respondents in support of human tissue donation (a), prior awareness of the UK Brain Bank Network (UBBN) (b), prior awareness of the use of pet brain tissue in research (c), support for the use of pet brain tissue in research (d), support for the establishment of the Companion Animal Brain Bank (CABB) (e) and likelihood of donation of own pet to the CABB (Fisher's exact or chi‐squared test)

As 87 (47%) respondents were associated with the veterinary profession, we then compared key responses between veterinary‐affiliated (VA) and non‐veterinary‐affiliated (NVA) individuals (summarised in Figure 1). The percentages reported are calculated from the number of respondents who answered the question. Of 68 VA individuals who provided a response, 64 (95%) supported human organ donation and four (5%) were unsure, compared with 66 of 79 (84%) NVA individuals who supported, eight (10%) were unsure and three (4%) did not support human organ donation (*p* = 0.123). Ten (15%) VA and 10 (13%) NVA individuals had previously heard of the UBBN (*p* = 0.632), while 49 (73%) VA respondents were aware of brain tissue from pets being used for human and veterinary research, compared with 45 (60%) NVA individuals (*p* = 0.115). Fifty‐nine (87%) VA individuals supported brain tissue from pets being used in research and 66 (87%) NVA individuals (*p* = 0.907). The four respondents who strongly disagreed or disagreed with brain tissue from pets being used in research were all NVA individuals. Sixty (77%) VA and 63 (82%) NVA individuals supported the establishment of the CABB (*p* = 0.745). Three (2%) respondents either strongly opposed or opposed the CABB; all NVA individuals. Forty (63%) VA and 50 (70%) NVA individuals were likely or highly likely to donate their pet's brain for research purposes (*p* = 0.270).

## DISCUSSION

The principal objective of this study was to assess pet owner perceptions of brain and tissue banking to support veterinary and translational neuroscience research. In so doing, we aimed to raise awareness of the CABB and to optimise effective engagement of clients who would consider donating their pet's brain tissue postmortem, with the view to using this information to improve the consent and donation process for companion animals.

The online survey was hosted via our institution's social media pages and a relatively small number of respondents took part (185), with a significant female bias (93%), as has been previously reported.^25^ All respondents were pet owners, and 47% were associated with the veterinary profession. Background information was provided at the beginning of the survey to ensure that respondents were informed of the existence and goals of the CABB. Interestingly, only 14% of respondents were aware of the UBBN's existence prior to this survey. Despite this, 87% of respondents supported the use of brain tissue from pets being used in research. The majority (90%) of respondents supported the development of the RVC CABB, believing that this project will benefit UK neuroscience research and positively influence perceptions of the institution overseeing the process. Major factors in the respondent's support of the CABB included the concept of their pets contributing to research after death, aiding other veterinary patients suffering from similar conditions but also by facilitating critical research into major neurological conditions in humans. Two respondents shared personal experiences of neurological diseases in their family, including frontotemporal dementia and tertiary neuroborreliosis, urging further research in these areas. In the USA, over 60% of people in one study indicated that they would be likely or somewhat likely to donate their brain for research.^22^ Individuals most likely to donate also indicated a higher willingness to participate in other neuroscience research projects.^22^ One responder in our study expressed interest in organ donation as an alternative to live animal experimentation, and the potential for archived brain tissue to reduce the number of animals used in research is an area that merits further research.

Two percent of responders opposed the RVC CABB and felt that it would negatively influence their opinion of the veterinary hospital involved. These respondents were also against the use of human and animal organ donation for disease‐based research. The most common concerns raised regarding a CABB were the desire to bury their pet or to cremate it intact (43%) and the desire to avoid any interventions/procedures on their pets postmortem (40%). Barriers to human brain donation are often centred around concerns and misconceptions about brain research and the process of brain donation.^26^ Accordingly, the willingness to donate has been shown to markedly increase when information on the process and purpose of brain donation is provided.^27^, ^28^ In one study, discussion of brain donation in the context of end‐of‐life treatment decisions resulted in an increase in donation rate from 2% to 29%.^29^ These findings highlight the importance of donor engagement and the provision of easily accessible and clearly written information on the process and purpose of donation.^22^, ^26^, ^27^, ^28^ In this study, summary information was provided to respondents on how the procedure is performed, what happens to the brain tissue and how their pet's body will be taken care of sensitively and respectfully. Following collection of the brain, the pet can be immediately transferred to a crematorium of the owner's choice for individual cremation, so CABB donation will not interfere with the option for owners to have their pet's ashes returned to them. Seven survey respondents commented that despite reading the information provided, they did not feel adequately informed about the CABB to make a decision regarding their willingness to donate their pet's brain. This highlights the need for the provision of clear and comprehensive information in written and verbal formats to reassure and educate owners on the processes involved. Based on the results of this survey, respondents would favour the provision of additional information via websites and in printed form at the veterinary hospital, with further clarification provided by conversation with the attending veterinarian at the time of donation. Improving access to information on the CABB was generally favoured by the respondents, with 94% agreeing that information on brain banking should be made more widely available to the public to inform those who may wish to pursue brain donation and to introduce this field of research to those currently unaware of its potential. Opinion on the optimal timing for gaining consent for donation to the CABB varied among respondents, with some favouring an open discussion when their pet remains well and others favouring such a discussion when their pet is showing declining health or at the time of euthanasia. In light of this variation in response, sensitively increasing awareness of the existence of the CABB at all stages of veterinary care (e.g., through promotional materials within the hospital) may be warranted, with direct client–veterinarian discussions then undertaken in consultations when the pet is diagnosed with neurological disease.

Eighty‐five percent of responders knew someone affected by a neurological disorder and 24% were aware of neurological conditions that affect their pet's breed; thus, an individual motivation or desire to advance the field of neurology to develop new diagnostic tools and therapies was commonly reported. However, 47% of respondents were associated with the veterinary profession and so are likely to have a greater exposure to and prior knowledge of neuroscience and neurological disease, as well as an inherent interest in veterinary research and desire to promote continued developments in animal health care provision. These demographics may have resulted in a bias towards a more positive response to a CABB and are not representative of the general pet owning public. That being said, when VA respondents were compared to all others, no statistically significant differences were identified in the responses to key questions. However, the four respondents who disagreed with the use of pet brain tissue for research purposes were not affiliated with the veterinary profession. Future studies to evaluate a larger population unaffiliated with the veterinary profession may help to explore reasons for disagreement with companion animal brain use in research, facilitating more in‐depth thematic analysis and ultimately contributing to the development of strategies to improve awareness and support of the CABB.

Our study has important inherent limitations that are likely to have impacted the results. The sample size was relatively small, potentially due to inadequate advertisement of the questionnaire, lack of an incentive to participate and/or low interest in participation. There was a significant female bias in the respondents and 47% of them were associated with the veterinary profession. The social media pages used to advertise the questionnaire predominantly reach clients, staff, students and others affiliated with the RVC; future studies targeting a broader population of pet owners across the UK would provide information on views of brain banking across other regions and institutions. The questionnaire did not request information such as family composition, involvement in animal welfare societies, animal breeding or other associated activities that may influence perspectives on CABB and interest in particular aspects of animal or neuroscience research. Furthermore, the goals and potential benefits of the CABB were described prior to the respondent completing the questionnaire and may have impacted the subsequent responses provided. However, the data generated provide important insights into the positive perspectives on companion animal brain banking in informed pet owners in the UK; following minimal information provision, we identified an overarching positive response to companion animal brain collection and archiving. These data can be used to adapt our current strategies to raising owner awareness and to obtaining consent. Future studies to identify pet owner perspectives both before and after being provided with detailed information on the CABB and studies of a larger, more diverse pet owner population would be beneficial.

Ultimately, the information gathered from this study will be used to tailor publicity, consent and procedural materials regarding the CABB. The data will also shape the critical and sensitive conversations that must take place between veterinarian and pet owner. While companion animal brain banking is inevitably a complex and relatively sensitive area, in this preliminary study we found pet owner perceptions to be positive and supportive of an open dialogue regarding the potential options for brain banking and the associated value to progressive neuroscience research and clinical advancement.

## CONFLICTS OF INTEREST

The authors declare they have no conflicts of interest.

## AUTHOR CONTRIBUTIONS

Thomas J. A. Cardy and Daniel Jewth‐Ahuja designed the study and collected the data. Thomas J. A. Cardy, Daniel Jewth‐Ahuja and Abbe H. Crawford analysed and interpreted the data and drafted and revised the manuscript.

## ETHICS STATEMENT

The authors confirm that the ethical policies of the journal, as noted on the journal's author guidelines page, have been adhered to. The research was approved by the Ethics and Welfare Committee at the Royal Veterinary College (approval number SR2017‐1327). Study participants provided full consent prior to undertaking the questionnaire.

## Supporting information

Supporting information.
Supporting Document 1: Royal Veterinary College's Companion Animal Brain Bank (RVC CABB) protocol summary
Supporting Document 2: Full questionnaire
Supporting Document 3: Royal Veterinary College's Companion Animal Brain Bank (RVC CABB) owner information sheet
Supporting Document 4: Royal Veterinary College's Companion Animal Brain Bank (RVC CABB) owner consent form

## Data Availability

Data available on request from the authors.
