# Supplementary material for: Perceptions and attitudes towards companion animal brain banking in pet owners: A UK pilot study
Source: Vet Rec Open. 2022 May 28;9(1):e36. doi: 10.1002/vro2.36 (PMC9142818; doi:10.1002/vro2.36)
Supplement: Supplementary file 1 — Supporting information. Supporting Document 1: Royal Veterinary College's Companion Animal Brain Bank (RVC CABB) protocol summary Supporting Document 2: Full questionnaire Supporting Document 3: Royal Veterinary College's Companion Animal Brain Bank (RVC CABB) owner information sheet Supporting Document 4: Royal Veterinary College's Companion Animal Brain Bank (RVC CABB) owner consent form [file VRO2-9-e36-s001.pdf]

SUPPORTING INFORMATION S1

Companion Animal Brain Bank (CABB), Royal Veterinary College
Protocol Summary

The objective of the CABB is to collect and store biological specimens for research and educational purposes. This has been made possible by the generous support of the Animal Care Trust.

Inclusion criteria:

- Any dog or cat being euthanised due to a neurological condition
- Additional dogs and cats being euthanised due to a non-neurological condition to provide control samples
- Owners must provide informed consent prior to euthanasia

Exclusion criteria:

- If more than 72hrs have elapsed between euthanasia and collection of the brain
- Failure to place the body in cold storage promptly after euthanasia
- Any animals that have received cytotoxic medication

Protocol:

- QMHA Neurology Team contacts the Clinical Investigation Centre (CIC) when a suitable patient is due to be euthanised or has been euthanised, and the owner has provided consent for donation of their pet to the CABB.
- Following euthanasia, the patient is immediately transferred to cold storage and a unique CABB number is assigned.
- A sample pack is collected from the CIC. The pack includes a sample log form, small polybags, cryovials containing 1ml RNALater, serum gel, plain and EDTA tubes
- The brain is removed from the calvarium as soon as possible after death (and within 72 hours). It is weighed, length measured and photographed, prior to sectioning down the sagittal midline. Two small (approximately 5mm by 5mm) sections of the frontal lobe are placed in RNALater. One hemibrain is placed into 4% paraformaldehyde for a minimum of 5 days, and a maximum of 12 days. The other hemibrain is sectioned into rostral, mid and caudal sections prior to snap freezing in liquid nitrogen and storage at -80°C.
- Residual serum, whole blood, CSF and urine samples were available are collected from the diagnostic laboratories and stored in up to 500μl alliquots at -80°C.
- RNALater samples are stored at 4°C for 12-24hrs before being transferred to -80°C.
- Following fixation, the hemibrain is sectioned from rostral to caudal into 3-5mm transverse sections, prior to routine processing through ethanol and xylene and embedding in paraffin wax. Paraffin-embedded sections are archived at room temperature.
- All stored samples are recorded in the CABB data base.

Histopathological analysis of paraffin-embedded sections of the lesion and other areas of interest is performed by the RVC Anatomical Pathology Service and a full report provided. Owners and referring veterinary surgeon's that wish to be informed of the histopathological diagnosis are informed by the attending RVC Clinician. There is no charge to the owner for this report.

Researchers in the UK and internationally wishing to utilise samples held in the CABB are requested to contact the CABB team via the CIC (cic@rvc.ac.uk). All requests will be reviewed for suitability, feasibility, and appropriate ethical approval by the CABB Team and CIC. Following approval, the requested samples will be provided free of cost (with the exception of shipping costs). Residual samples should be returned to the CABB following completion of the proposed work.

SUPPORTING INFORMATION S2

SURVEY
Introduction to the Companion Animal Brain Bank (CABB)

Introduction

- Neurological diseases are those that affect the brain, spinal cord or nerves. They cause severe and debilitating symptoms in humans and companion animals such as dogs and cats, resulting in significant lifelong challenges for carers and owners.
- Many brain diseases currently have no effective treatment and research is restricted by a lack of available brain tissue to study.
- This restriction is being overcome by establishing the UK Brain Bank Network in human medicine
- Brain Banks are a secure store where samples of brain tissue are kept after a patient has died or been euthanised. Researchers in human and veterinary medicine can gain access to this tissue to help them in their research
- Companion animals that are euthanised at **The Royal Veterinary College (RVC)** due to brain diseases are often cremated without post-mortem examinations to determine a diagnosis. This means that these brains are never available for research.

Mission statement

The Royal Veterinary College is setting up a **Companion Animal Brain Bank** with the goals of:

- Routinely collecting brain tissue from dogs and cats that are euthanised because of brain disease.
- Allowing veterinarians and owners to obtain a more accurate diagnosis of their pet's brain disease.
- Providing dog brain tissue to human and veterinary researchers across the UK.
- Allowing researchers to gain a better understanding of brain diseases at a cellular and molecular level.
- Providing comparisons between human and dog brain diseases to better understand how brain diseases work. In turn this could lead to novel or improved treatments for pets and humans.

Owner Consent Statement

Thank you very much for agreeing to participate in this survey.
The information provided by you in this questionnaire will be used for research purposes. It will not be used in a manner which would allow identification of information about yourself, your pet or your individual responses.
Anonymised research data will be securely archived at the Royal Veterinary College in order to make them available to other researchers in line with current data sharing practices.

1. I confirm that I have read and understand the information sheet for the above study ☐
2. I understand that my participation is voluntary and that I am free to withdraw at any time, without giving reason ☐
3. I agree to take part in the above study ☐

General client information

Taking part in this survey is entirely voluntary and we greatly appreciate your time and interest in participating. You are under no obligation to answer all the questions. The data obtained from your answers will solely be used for research purposes and shall remain confidential. This information has the potential to be used in scientific publications whereby the identity of you and your pet will continue to remain anonymous in all published and written data.

This project has been reviewed and approved by the Royal Veterinary College Research & Ethical Review Board (SR2017-1327).

What is your age?

- 18 – 24
- 25 – 34
- 35 – 44
- 45 – 54
- 55 – 64
- 65 and over

What is your gender?

- Female
- Male
- Other (with an empty box to write in)
- Prefer not to say

What is your religion (if any)?

- Christian
- Muslim
- Jewish
- Buddhist
- Hindu
- Sikh
- Greek or Russian Orthodox

- Atheist
- Agnostic
- Other

What is your occupation? (blank box to be filled in)

What country do you live in? (drop down list with countries)

What is your current level of education? (please tick most recent qualification)

- School
  - GCSEs
  - A-Levels
  - BTEC
  - International Baccalaureate
- Degree
- Post grad (PhD, Masters etc.)
- Prefer not to say

Have you ever visited the Queen Mother Hospital for Animals (QMHA)?

- Yes
- No

If yes, how long have you been a client at the Queen Mother Hospital for Animals for?

What pets do you have? (tick boxes with dog, cat, exotics, horse, livestock)

<u>Patient information</u>

This section focuses on specific information regarding your pet.

Diseases of the brain are influenced by many factors including gender of the pet, age and breed. This is particularly the case for brain diseases and so we are interested to find out as much as possible about your pet.

What breed is your dog/cat?

How old is your dog/cat?

What sex is your dog/cat?

- Male
- Female

Is your dog/cat neutered/spayed?

- Yes
- No

Have you bred from your dog/cat?

- Yes
- No

Have you ever bred a litter of puppies/kittens before?

- Yes
- No

Is your dog/cat currently insured?

- Yes
- No

Has your pet ever previously visited any of these services at the Queen Mother Hospital for Animals?

- First Opinion Out of Hours Emergency Clinic
- Dermatology
- Emergency & Critical Care
- Internal medicine
- Oncology
- Ophthalmology
- Orthopaedic Surgery
- Physiotherapy
- Soft Tissue Surgery
- Sports Medicine Osteoarthritis and Pain clinic

Does your dog/cat currently suffer from any illness?

- Yes
- No
- If yes, please specify:
    - Neurological (brain, spinal cord, nerves, eyes)
    - Cardiovascular (heart)
    - Dermatological (skin and hair)
    - Gastrointestinal (stomach and intestines)
    - Musculoskeletal (muscles and joints)
    - Reproductive system
    - Respiratory (lungs)

Has your vet ever taken note of any of these symptoms in your dog/cat?

- Behaviour changes
- Blindness
- Circling
- Head tilt
- Imbalance
- Incoordination
- Seizures
- Tremors
-

Do you know of any pets belonging to friends, family or work colleagues with brain disease?

- Yes
- No

Are you aware of any brain diseases that affect your dog/cat's breed?

- Yes
- No

Organ Donation

This section focuses on the topic of organ donation in humans. A donation is where a person donates their organs and tissues for transplant to someone else or for use in research. Organ donation helps save thousands of lives in the UK every year and is also critical for research in a variety of diseases. Anyone can register to donate their organs and tissue when they die, regardless of their age or medical conditions.

Do you support human organ donation?

- Yes
- No
- If yes, which of these do you support the donation of:
    - Bone
    - Cornea
    - Heart
    - Kidney
    - Liver
    - Lung
    - Pancreas
    - Small bowel
    - Tissue (skin, eyes, heart valves, arteries)

Are you on the NHS Organ Donor Register?

- Yes
- No

Do you support the donation of organs for research purposes?

- Yes
- No

Are you in favour of the ''Opt-out'' scheme for organ donors? (The Opt-out scheme is a system where you would automatically become a donor unless you specifically state that you do not want to donate.

- Yes
- No

Do you believe there should be more choice in this scheme?

- Yes
- No

Pet neurology and the UK Brain Bank Network

The UK Brain Bank Network is an initiative directed by the Medical Research Council (MRC) to establish a coordinated network of brain tissue collection sites (banks) across the UK. Members of the public donate the brains after their death. Donated brains enable accurate diagnosis of the disease and important research into brain diseases that affect thousands of people every year including:Alzheimer’s disease, Depression, Parkinson’s disease, Schizophrenia, Stroke and Multiple Sclerosis.

Were you aware of the UK Brain Bank Network prior to this survey?

- Yes
- No

Has anyone you know ever been diagnosed with a brain disease?

- Yes
- No

Are you aware that brain tissue from pets can help with human as well as veterinary research?

- Yes
- No

Do you support the idea of donating organs from dogs and cats for research?

- Very strongly agree
- Strongly agree
- Agree
- Neither agree nor disagree
- Disagree
- Strongly disagree
- Very strongly disagree

Do you support the usage of animal tissue in research for human diseases?

- Very strongly agree
- Strongly agree
- Agree
- Neither agree nor disagree
- Disagree
- Strongly disagree
- Very strongly disagree

Do you support the usage of animal tissue in research for veterinary diseases?

- Very strongly agree

- Strongly agree
- Agree
- Neither agree nor disagree
- Disagree
- Strongly disagree
- Very strongly disagree

Royal Veterinary College Companion Animal Brain Bank

Routine collection of brain tissue from pets has potential benefits for owners, veterinarians, and the research community. Collected brain tissue can provide a more accurate diagnosis of the causative brain disease. It also allows research into brain diseases in pets and humans so improving understanding and potentially leading to improved treatment. Collected brain tissue will allow us to provide more information to pet owners about their pet's disease whilst helping with research into brain diseases.

Removal of brain tissue takes place after your pet is put to sleep (termed 'euthanasia'). The procedure requires you to provide written consent and is free of charge. After euthanasia, your dog would be transferred to an area where the brain is removed and processed. Key details are recorded by an experienced technician such as age, breed, sex, body weight, reason for death/euthanasia, date of death/euthanasia and suspected diagnosis. All details will also be added to an online database where the information is anonymised and securely stored. All procedures will be carried out in a humane and sensitive fashion in line with current hospital guidelines and working practices.

Due to the nature of this procedure, you will be not be able to receive your pet's body for burial, however, you will still be able to have the option to have them individually cremated (with their ashes then returned to you) if you choose so.

Would you support the Royal Veterinary College in establishing a Companion Animal Brain Bank?

- Yes
- No

Would the presence of a Companion Animal Brain Bank at the Royal Veterinary College positively or negatively affect your perceptions of the Queen Mother Hospital?

- Very strongly positive
- Strongly positive
- Positive
- Neither positive nor negative
- Negative
- Strongly negative
- Very strongly negative

Do you believe the Companion Animal Brain Bank will benefit veterinary and human research?

- Very strongly agree
- Strongly agree
- Agree
- Neither agree nor disagree
- Disagree

- Strongly disagree
- Very strongly disagree

In the highly unfortunate circumstance of your pet being euthanised (put to sleep), would you give consent to donate his/her brain tissue for research?

- Strongly agree
- Agree
- Neither agree nor disagree
- Disagree
- Strongly disagree

If you support the idea of a Companion Animal Brain Bank being established at the Royal Veterinary College, what are your reasons? (select as many as apply)

- I like the idea of my pet being able to help researchers understand brain diseases better
- I know of someone who is currently suffering from a brain disease and thus understand the importance of improving research in this field
- I think that after my dog has passed away, they will not need their brain anymore
- Knowing how the UK Brain Bank Network has improved research into human diseases I believe the Companion Animal Brain Bank will bring a similar level of progress to veterinary research
- It is a procedure which is free of charge
- Other (please specify)

Are there any reasons that make you less likely to give consent for your pet to donate his/her organs after death?

- This is against my religion
- It is against my personal ethics
- Lack of information on the procedure
- I would like my pet to be buried or cremated intact
- I would not like for further procedures to take place after my pet's death
- I do not like the thought of it
- My pet cannot consent to this procedure
- I do not like the idea of my pet's brain being used for research purposes
- Other (please specify)

To participate in the Companion Animal Brain Bank it is required that an owner provides written consent. When do you feel the best time for an owner to provide consent is?

- When the pet is euthanised
- When the pet is diagnosed with a brain disease
- When it is clear that the pet's health is deteriorating
- Other (please specify)

As a pet owner, what would be your preferred method of receiving information about the Companion Animal Brain Bank?

- Email
- Mail
- Pamphlets being made available at the Queen Mother Hospital

- Being told by the veterinary surgeon seeing your pet
- Other (please specify)

Do you believe information on initiatives such as the UK Brain Bank Network and Companion Animal Brain Bank should be made more readily available to the public, to raise awareness of brain donation for human and veterinary research?

- Very strongly agree
- Strongly agree
- Agree
- Neither agree nor disagree
- Disagree
- Strongly disagree
- Very strongly disagree

SUPPORTING INFORMATION S3

Owner Information Sheet for Body Donation

Dear Pet Owner,

We understand that losing a pet is a difficult time for all involved; we are truly sorry for your loss.

Here at the Royal Veterinary College (RVC), we have established a Companion Animal Brain Bank, collecting samples of brain tissue, blood and other biological specimens. Donating your pet's tissues is a valuable gift - your generous donation will become an important resource for training veterinary professionals and for research.

What is the purpose of the bank?

The objective of the bank is to collect and store biological samples for research and educational purposes. Researchers can use the stored samples for future research projects to learn more about diseases in companion animals. Having samples from many pets allows researchers to identify trends and discover better ways to diagnose, prevent and treat many conditions. Your pet's samples will also provide valuable genetic material (DNA, RNA) which researchers can use to learn more about the role genes play in health and disease.

What will the bank collect and store?

In addition to samples taken as part of the diagnostic investigation, we would also like to keep brain tissue and biological samples collected following the euthanasia of your pet.

Will I be contacted about future research?

Neither the RVC nor researchers who access samples or information from the bank will contact you. If you would like to keep informed about ongoing research projects at the RVC please like the *Clinical Investigation Centre – RVC Facebook Page*

How will the bank share samples and information with other researchers?

Researchers may contact the bank to request permission to use your pet's samples and health information for their studies. An ethical review board will consider the researchers qualifications and proposed research and will determine if any additional review or approval is necessary.
The bank will allocate your pet a unique reference number, which will identify your pet's samples and health information. The bank removes any reference to your identity and this information remains anonymous to third parties.

Does donating to the bank cost anything?

There will be no additional charge for donating you pet's body to the bank.

Will I receive any rewards for donating?

There will be no payment for donation of tissues or health information. The samples donated will become the property of the RVC. However, they may lead to new medical knowledge, treatments or products. These products will not be associated with any financial reward to yourself.

Will I receive individual results from research performed using my pet's samples?

Unfortunately, as all samples are anonymised we are unable to provide individual results at this time.

Thank you for your consideration in donating your pet's body. These donations are highly valued by staff and students at the RVC. Great care is taken to ensure that your beloved pet is treated with dignity from the moment your pet is entrusted in our care.

SUPPORTING INFORMATION S4

Companion Animal Brain Bank – Body Donation

OWNER'S CONSENT FORM

PLEASE COMPLETE IN BLOCK LETTERS:

Animal's Name:		Owner's Surname:	
Species:		Breed:	
Date of Birth:		Gender/Neuter status:	

I, the undersigned, acknowledge that I am the (owner or authorised agent of the owner) of the above named animal and that I am 18 years or older. I have read the owner information sheet, entitled "Owner Information Sheet for Body Donation" and that any questions that I presently have regarding the body donation, have been adequately answered. I declare that I fully agree to allow the release of the body of my pet (identified above) including fluids such as cerebrospinal fluid, blood, serum, plasma and urine. I understand that my pet will be euthanised by my veterinary surgeon and the body will be used for education at the Royal Veterinary College (RVC) and/or for research purposes. I give permission for my pets remains to be cremated according to my wishes.

I have been made aware that I have certain rights under the data protection laws. I give my consent to the collection, processing, disclosure and transfer (including transfers to persons outside the European Economic Area (EAA)) of my personal data for the purposes of the administration of this research and for regulatory requirements. I understand that I will not be referred to by name or otherwise identified in any report or publication. I confirm that I do not wish to restrict the use of personal data or results which arise from this research in any way, save where this conflicts with my rights under any relevant data protection laws.

I am aware that the RVC are unable to provide me, or any other person, with specific information regarding the use of my pet's body. I understand that specimens of tissue, including the brain, may be retained for future use in teaching and/or research.

I accept that samples and images may be shared with third parties and used in approved research projects by the RVC and its collaborators. I agree that the RVC shall be free to publish the results of these projects. I give permission for my pet's health history to be given to the RVC in order to enhance learning and understanding.

________________________ ____________________

Signature of Owner Date

I have explained the contents of this letter to the above client.

________________________ ____________________

Witness Veterinarian Signature Date

Thank you very much for your support – your generosity allows us to train future vets, improve understanding of diseases and develop better treatment for pets.
